# Barriers Are Not the Limiting Factor to Participation in Physical Activity in Canadian Seniors

**DOI:** 10.1155/2012/890679

**Published:** 2012-09-04

**Authors:** Kristy L. Smith, Kelly Carr, Alexandra Wiseman, Kelly Calhoun, Nancy H. McNevin, Patricia L. Weir

**Affiliations:** Department of Kinesiology, University of Windsor, Windsor, ON, Canada N9B 3P4

## Abstract

The identification of barriers to physical activity and exercise has been used for many decades to explain exercise behavior in older adults. Typically health concerns are the number one barrier to participation. Data from CCHS-HA dataset (*N* = 20, 875) were used to generate a sample of Canadians, 60+ years, who did not identify a health condition limitation, illness, or injury as a barrier to participation in physical activity (*n* = 4,900) making this dataset unique in terms of the study of barriers to participation. While the vast majority of older adults participated in physical activity, 9.4% did not. The relationships between nonparticipation, barriers, self-reported health status, and chronic health conditions were determined using binary logistic regression. The main findings suggest that traditional barriers and self-reported health status are not responsible for nonparticipation. Nonparticipation was best predicted by chronic health conditions suggesting a disconnect between self-reported health status and underlying health conditions. The data are clear in suggesting that barriers are not the limiting factor and physical activity programming must be focused on meeting the health needs of our aging population.

## 1. Introduction

The world's population is aging. In 2009, 14% of Canada's 32 million people were aged 65 years or older. This proportion is expected to rise to between 23 and 25% by 2036; effectively doubling the number of seniors observed in 2009 [1], placing increased demand on the health care system and the nation's workforce. With this dramatic demographical change in the population, it has become increasingly important to find effective ways to improve health and prolong independence among older adults. Participation in regular physical activity can provide numerous physiological, cognitive, and psychological health benefits in the aging population. While some level of decline, such as reduced muscle strength and slower responding, is a normal part of the aging process [2], there is evidence that habitual exercise can minimize the physiological effects of an otherwise sedentary lifestyle and prolong active life expectancy [3]. However, despite this knowledge, many individuals do not engage in recommended levels of regular physical activity [4], and the Seniors in Canada report card [5] gave Canadian seniors a grade of C+ for participation in physical activity, with 62% of seniors being inactive.

The sizeable proportion of inactive individuals forces a critical examination of the unique challenges faced by older adults, aimed at developing the most effective health strategies to promote physical activity in this cohort [6]. Efforts to optimize health in this manner have led to numerous studies focused on identifying barriers and motivators to exercise among the older adult population, and several determinants have been consistently identified in the research literature as significant factors related to exercise behaviour among older individuals [7]. Considerations such as health concerns [6, 8, 9], lack of knowledge [6, 10], fear of falling or injury [6, 9, 11], time constraints [6, 8, 9], and lack of transportation [6] are generally reported as disincentives to participation by older adults. 

Typically, issues contributing to the lack of participation are elicited through the use of focus group discussions [6, 9], interviews [11], or questionnaires [12]. These studies can be complex, such that some issues such as health problems are highly related to each other and can function both as a barrier and a motivator to exercise [6, 8]. In addition, the aging population is a diverse group [9, 13], with no one common barrier reported across all racial and ethnic groups [9]. Level of mobility [12], current exercise status [9], and past experiences [11] can all influence what respondents identify as posing a barrier or an enabler to physical activity. 

The majority of older adults do, however, identify at least one of these considerations. In the cohort studied by O'Neill and Reid [14], 87% of the elderly respondents identified at least one barrier that prevents them from engaging in exercise. While the most common barriers appear to be related to health concerns, there are members of the sedentary population that do not identify any barriers to physical activity participation. For example, Cohen-Mansfield et al. [8] stated that 89 of their 324 person sample either reported no barriers at all or that they already engaged in exercise. Ironically, Connell et al. [15] reported that good health itself was a barrier to exercise because it provided the older adults with no reason to exercise. 

 The goal of the current study was to identify the relationship, if any, between what older adults perceived as barriers to physical activity and participation. Because previous research has suggested that health concerns pose a significant barrier to participation and would thus likely skew the data towards health-related barriers, we chose to focus on a subset of older adults who reported having no health condition limitation, illness, or injury that prevented them from participating in physical activity. We further delineated the analyses by gender as it has been well documented that older women tend to report more chronic diseases than men and this difference may impact overall participation rates [16–18]. Using data from the Canadian Communities Health Survey-Healthy Aging, an overall sample size of 4,900 seniors was identified, and, of these, 9.4% reported no participation in physical activity over the last seven days. We examined the relationship between their nonparticipation and barriers to participation, self-reported health, and chronic health conditions, and hypothesized that traditional barriers would not predict nonparticipation and there may be a dissociation between self-reported health status and reported chronic health conditions. The overarching goal of this study was to identify why Canadian seniors who do not report a health limitation do not participate in regular physical activity. 

## 2. Methods

### 2.1. Canadian Communities Health Survey-Healthy Aging (CCHS-HA)

Data from the Canadian Community Health Survey-Healthy Aging (CCHS-HA) (Statistics Canada, [19]) were used for the current study. This cross-sectional survey gathered self-reported data on the “factors, influences and processes that contribute to healthy aging through a multidisciplinary approach focusing on health, social and economic determinants” [19, page 1]. CCHS-HA data were derived through a standardized interview of adults aged 45 years of age and greater from the ten provinces in Canada, between December 2008 and November 2009. Using computer-assisted personal interviewing (CAPI), trained field interviewers conducted 94% of the interviews face-to-face, with the remainder being conducted over the telephone. The objectives of the survey were to (1) better understand the aging process; (2) examine how lifestyle impacts health; (3) use a multidisciplinary approach to examine relationships between healthy aging and geographical, social, demographic, and economic factors; and, (4) provide information on successful aging. 

### 2.2. Participants

For the purposes of our study, we only used responses from the CCHS-HA dataset for people aged 60 years and older (*N* = 20,875). Given the interest in identifying barriers to participation, we further limited our sample to those seniors who did not identify a “health condition limitation” or “illness or injury” as barriers to participation. This ensured that we were not examining individuals who did not believe they were physically able to participate. Master weights were applied to the overall dataset such that the weights had a mean of 0. The weighting value for each respondent corresponded to the number of persons in the entire population that the respondent represented. The final weighted sample size was 4,900 participants (23.47% of total *N*; male: *n* = 2183; female: *n* = 2717). To estimate the average age of participants, the mid-point of each age-range was used (e.g., 60–64 years = 62.5). For the 85+ age range, we used 87.5 years, which may result in a slight underrepresentation. The overall average age of the sample was 68.39 ± 6.91 years.

### 2.3. Variables of Interest

#### 2.3.1. Participation

This was determined using the derived variable for participation in leisure physical activities. This categorical variable indicated whether the respondent had participated in walking for pleasure or exercise, light sports, moderate sports, strenuous sports, and exercises to increase muscle strength and endurance over the 7 days prior to the interview. It was scored as either a “1” indicating reported participation over the last 7 days, a “2” indicating no participation over the last 7 days, or a “9” indicating at least one required activity had not been responded to (these were excluded from future analyses). To confirm differences in participation, the PASE (Physical Activity for the Elderly Scale) score was also examined [20]. The PASE score combines information on the frequency and duration of participation in leisure, household, and occupational activity over a seven-day period. While it does not differentiate frequency and duration, a higher score indicates greater levels of participation.

#### 2.3.2. Barriers to Participation

Thirteen barriers to participation were listed in the CCHS-HA dataset: cost, transportation problem, not available in area, location not physically accessible, location is too far, health condition limitation, illness or injury, fear of injury, lack of time, lack of energy, lack of motivation, lack of skills or knowledge, other. Similar to the participation measure, these were categorical variables where respondents indicated that “yes” it prevented participation, or “no” it did not prevent participation. Of these thirteen variables, ten were used to predict participation. Health condition limitation, illness or injury were used only to identify the current sample, and “other” was not included as it was not possible to draw a conclusion about illness, or injury from this response. The total number of barriers was derived (range: 0–8 barriers; mean = 1.06 ± 0.72 barriers; median = 1.00) from the number of barriers each participant responded “yes” to.

#### 2.3.3. Self-Reported Health Status

Respondents were asked “In general, would you say your health is” excellent, very good, good, fair, poor. This categorical variable was recoded such that excellent and very good were collapsed together, and fair and poor were collapsed together leaving three levels of self-reported health status.

#### 2.3.4. Chronic Health Conditions

The CCHS-HA included a number of self-reported chronic condition variables that were coded categorically. The following seven were used in the current study: (1) vision function—this variable was derived from five items based on the respondent's ability and/or inability to see well enough to read newsprint and be able and/or unable to recognize a friend on the other side of the street, with and/or without glasses or contact lenses. This resulted in three vision categories: no vision problems, problems corrected by lenses, and problems not corrected by lenses. No vision problems and corrected vision were collapsed together resulting in the two categories of no vision problems and vision problems; (2) heart disease—if the respondents reported having either angina or a heart attack, they were considered to have heart disease; (3) chronic obstructive pulmonary disease (COPD)—if the respondent reported having been diagnosed with chronic bronchitis, emphysema, or COPD, which yielded a broad category used to describe limitations in lung airflow; (4) diabetes; (5) osteoporosis; (6) living with the effects of a stroke—this was used to reflect neurological damage; (7) mobility trouble—this variable was derived from five items based on the respondent's ability to walk a short distance and/or around their neighborhood, with and/or without the assistance of another person and/or walking equipment and/or a wheelchair. This resulted in four mobility categories: no mobility problems, mobility problems—no assistance required; mobility problems—requires wheelchair; mobility problems—requires help/cannot walk. The mobility problems were collapsed together resulting in two mobility categories.

### 2.4. Data Analyses

Logistic regression techniques were used to examine the relationships between nonparticipation and barriers to participation, self-reported health status, and the seven chronic health conditions. Data were analyzed separately for male and female respondents. Odds ratios and 95% confidence intervals were used to identify the risk of nonparticipation as a function of the predictor variables. Due to the small sample size of nonparticipants (*n* = 459), results associated with a large range in confidence intervals should be interpreted with caution as they may overestimate the practical significance. SPSS 19 was used for all data analyses, with *P* < 0.05. 

## 3. Results

Table 1 reveals that a majority of the Canadian seniors in this sample were participants in physical activity over the last 7 days. While a small percentage of the respondents had not been physically active, this is the group of interest. Given that none of these respondents has identified barriers due to a health condition limitation, illness, or injury, the data show that approximately 10% do not participate in physical activity. Lower participation in the nonparticipants was confirmed through a lower PASE score (139.68) as compared to participants (142.99). Overall, the majority of the respondents report excellent/very good health, and a large majority do not suffer from the identified health conditions. 

As can been seen in Table 2, approximately 89% of participants, regardless of participation level, did not identify a barrier. When the barriers were used to predict nonparticipation, the results of the logistic regressions suggested that older males and females have different barriers to participation (see Table 3). Males were more likely to be nonparticipants as a function of the availability of the activity (OR = 4.50, CI = 1.09, 18.68), while females were more likely to be a nonparticipants due to a lack of time (OR = 1.45, CI = 1.09, 1.92). Nonparticipation was not related to a lack of motivation in males (OR = 0.69, CI = 0.48, 0.98) and was not related to a lack of energy in females (OR = 0.54, CI = 0.39, 0.76). Overall, the data suggest that barriers do not appear to be responsible for the nonparticipation of Canadian seniors.

Given that barriers were not strong predictors of nonparticipation, we examined the influence of self-reported general health status. As seen in Table 1, the distribution of respondents across the three levels of health did vary between participants and nonparticipants. Participants more frequently reported their health as being excellent/very good (61.2% versus 49.2%), and fewer participants reported their health as fair/poor (9.1% versus 13.5%). While these differences in frequency existed, for both males and females there was no relationship between self-reported health status and nonparticipation in physical and leisure activities (see Table 4).

With regards to chronic health conditions, males and females identified a different number of predictors to nonparticipation (see Table 5). For males, who identified three predictors, the greatest likelihood of nonparticipation was predicted by vision function (OR = 3.06, CI = 1.30, 7.21), followed by mobility troubles (OR = 2.65, CI = 1.01, 6.95), and diabetes (OR = 1.66, CI = 1.12, 2.44). Women identified four significant predictors of nonparticipation; mobility troubles (OR = 2.71, CI = 1.70, 4.31), COPD (OR = 2.11, CI = 1.34, 3.32), diabetes (OR = 1.85, CI = 1.30, 2.63), and heart disease (OR = 1.54, CI = 1.19, 1.99). 

## 4. Discussion

The purpose of this study was to examine the relationship between barriers to participation, self-reported health status, and chronic health conditions on nonparticipation in physical activity in Canadian seniors aged 60+ years who did not identify a health condition limitation, illness, or injury as a barrier to participation. Across both participants and nonparticipants, respondents on average identified 1.06 ± 0.72 barriers to participation in physical activity, with 89% of the respondents identifying no barriers to participation. 

The barrier that had the highest likelihood of predicting nonparticipation differed between males and females. Males were more likely to be nonparticipants due to the activity not being available in their area, although this needs to be interpreted with some caution given the small sample size. In contrast, women were more likely to be nonparticipants due to time. Time being the most significant barrier to nonparticipation in women is supported by earlier work by Yoshida et al. [21] and Johnson et al. [22]. However, it is in contrast to the findings of O'Neill and Reid [14], where respondents ranked time as the 14th barrier to participation. The participants in O'Neill and Reid also identified “I get tired easily” as the second most frequent barrier. In the current study, a lack of energy was not related to nonparticipation in women. 

Self-reported health status was not related to nonparticipation in the current sample. This may not be surprising given that this sample represented only those who did not identify a health condition, illness, or injury as barriers to participation. By limiting the sample to those who perceived their health as not posing a limiting factor, it is difficult to directly compare the results to other studies where health was consistently identified as the number one barrier to participation. What is surprising in the current study, however, is the relationship between chronic health conditions and nonparticipation. These results suggest that while Canadian seniors have underlying chronic health conditions they do not always identify them as being associated with their self-reported general health, nor do they view them as limiting conditions to participation in physical activity. This potential disconnect between self-reported health status and chronic health conditions is interesting, as heart disease, vision, COPD, diabetes, and mobility trouble increased the likelihood of nonparticipation across males and females. It is possible that these older adults have learned to compensate for these chronic health conditions in their everyday life and therefore no longer consider them barriers to participation nor consider them impacting their self-reported general health status. It is clear, however, that there are limiting factors to participation. Although health concerns have been considered motivators to participation [23], this does not seem to be the case in the current study.

These findings have implications for physical activity programming for seniors. While only a small number of older Canadians were identified who did not participate, this sample represented those who believed they did not have limitations to participation; effectively creating a “healthy” sample of older adults. Regardless of their self-perceptions, this group of seniors was less likely to participate because of underlying health conditions. This would suggest that programming should target the specific needs of older adults; whether that takes the form of activities and classes designed specifically for those with targeted health conditions, or increased knowledge of how to integrate and attract those with health conditions into preexisting classes. What is clear is that the results identify a dissociation between self-perceptions of health and the reasons older adults are at greater risk for nonparticipation. 

This study is not without limitations. The derived variable we chose from the CCHS-HA dataset captures physical activity as any one of walking for pleasure or exercise, light sports, moderate sports, strenuous sports, and exercises to increase muscle strength and endurance. While this variable is all encompassing for physical activity, it does not include activities of daily living, which may also be considered by some to contribute to their daily physical activity levels. Second, the self-reporting of general health status does not take into account the fact that many older adults learn to accommodate health concerns and to compensate for their impact on daily activities. Thus, a physical performance based measurement of health status could quite possibly have produced a different pattern of findings. Third, the chronic health conditions selected were done so to represent an overall picture of health-related issues that may impact participation. Specifically, they were chosen to represent vision, respiratory function, cardiovascular health, neurological health, diabetes, and mobility. It is by no means an exhaustive list of all chronic health conditions; any of which could impact ones' ability to participate in physical activity. The chronic health conditions were not validated in any way through performance measures, suggesting that their presence may have been under- or overrepresented in the self-reporting. Fourth, in predicting nonparticipation, the overall sample of nonparticipants represented only 9.4% of the total sample derived. Thus, the potential for type I error may be increased. Lastly, it was not possible to determine whether participants were meeting the recommended guidelines for participation in physical activity. While the PASE scores confirmed this group was more active than the nonparticipants, it was not possible to determine if this was due to frequency of participation or the duration of participation.

Overall this paper raises the issue that despite having created a “healthy” sample of older Canadians, there are still those who do not participate in regular physical activity. The 9.4% of seniors in this sample who did not participate is substantially smaller than the national average of 62%. However, it is important to note that this sample has been derived from older adults who reported no health condition or limitation preventing them from participating and thus is not representative of the general population of older adults. As such, their nonparticipation is not related to the presence of barriers such as opportunity or desire, but to specific chronic health conditions, and suggests a potential disconnect between self-perceived health and actual health status. Considering that only one-third or less of older Canadians achieve the recommended guidelines for leisure time physical activity [4], it is important to shift the focus from simply describing the barriers and motivators to physical activity to working towards designing programs that will address the health conditions facing older Canadians. With the changing age demographic, it will be important to encourage all older adults to participate in some form of physical activity on a daily basis, including a renewed focus on the role that activities of daily living can play in achieving fitness goals. One positive note to take from our findings is that 90% of the healthy older adults identified do engage in some form of physical activity, suggesting that public health messaging is reaching its mark. 

## Figures and Tables

**Table 1:** Sample descriptive statistics (weighted sample).

Variable	Category	*N*	%
Participation	Participant	4438	90.60
	Nonparticipant	459	9.40

PASE	Participant		142.99
	Nonparticipant		139.68

Gender	Male	2180	44.50
	Female	2717	55.50

	Excellent/very good	2944	60.10
	Participant	2715	61.2
	Nonparticipant	226	49.2
	Good	1491	30.40
Self-reported health status	Participant	1320	29.7
	Nonparticipant	171	37.3
	Fair/Poor	465	9.50
	Participant	404	9.1
	Nonparticipant	62	13.5

	Vision function		
	Yes	66	1.40
	No	4806	98.60
	Heart disease		
	Yes	2212	45.20
	No	2676	54.80
	COPD		
	Yes	231	4.70
	No	4664	95.30
	Diabetes		
Chronic health conditions	Yes	595	12.10
	No	4303	87.90
	Osteoporosis		
	Yes	641	13.10
	No	4253	86.90
	Effects of a stroke		
	Yes	63	1.30
	No	4837	98.70
	Mobility trouble		
	Yes	149	3.10
	No	4748	96.90

**Table 2:** Barriers by participation (weighted sample).

Variable	Category	*N*	%
	Participant		
	Yes	308	6.9
Cost	No	4130	93.1
	Nonparticipant		
	Yes	26	5.7
	No	433	94.3

	Participant		
	Yes	147	3.3
Transportation	No	4291	96.7
	Nonparticipant		
	Yes	15	3.3
	No	444	96.7

	Participant		
	Yes	283	6.4
Not available in area	No	4156	93.6
	Nonparticipant		
	Yes	14	3.1
	No	445	96.9

	Participant		
	Yes	50	1.1
Not physically accessible	No	4389	98.9
	Nonparticipant		
	Yes	4	0.9
	No	455	99.1

	Participant		
	Yes	173	3.9
Location is too far	No	4266	96.1
	Nonparticipant		
	Yes	8	1.7
	No	451	98.3

	Participant		
	Yes	76	1.7
Fear of injury	No	4363	98.3
	Nonparticipant		
	Yes	5	1.1
	No	454	98.9

	Participant		
	Yes	1922	43.3
Lack of time	No	2517	56.7
	Nonparticipant		
	Yes	184	40.1
	No	275	56.9

	Participant		
	Yes	456	10.3
Lack of energy	No	3982	89.7
	Nonparticipant		
	Yes	72	15.7
	No	387	84.3

	Participant		
	Yes	1281	28.9
Lack of motivation	No	3158	71.1
	Nonparticipant		
	Yes	146	31.8
	No	313	68.2

	Participant		
	Yes	34	0.8
Lack of skills or knowledge	No	4404	99.2
	Nonparticipant		
	Yes	4	0.9
	No	454	99.1

**Table 3:** Odds of nonparticipation as a function of type of barrier.

Barrier	Males		Females	
	Odds ratios	95% CI	Odds ratios	95% CI
Cost	2.05	0.73, 5.74	1.07	0.67, 1.71
Transportation	0.89	0.16, 5.04	1.03	0.56, 1.88
Not available in area	4.50*	**1.09**,** 18.68**	1.50	0.81, 2.78
Location not physically accessible	0.33	0.05, 2.31	1.53	0.39, 5.94
Location is too far	11.96	0.46, 313.09	1.70	0.76, 3.80
Fear of injury	2.29	0.25, 21.50	1.59	0.58, 4.34
Lack of time	0.79	0.56, 1.13	1.45*	**1.09**,** 1.92**
Lack of energy	0.91	0.56, 1.48	0.54*	**0.39**,** 0.76**
Lack of motivation	0.69*	**0.48**,** 0.98**	1.18	0.88, 1.58
Lack of skills/knowledge	0.75	0.07, 8.09	0.78	0.25, 2.40

^∗^ *P*<0.05.

**Table 4:** Odds of nonparticipation as a function of self-rated general health status.

Barrier	Males		Females	
	Odds ratios	95% CI	Odds ratios	95% CI
Excellent/very good	0.74	0.45, 1.20	0.74	0.45, 1.20
Good	0.78	0.46, 1.30	0.79	0.46, 1.30
Fair/poor	1.00	Referent	1.00	Referent

**Table 5:** Odds of nonparticipation as a function of health condition.

Barrier	Males		Females	
	Odds ratio	95% CI	Odds ratios	95% CI
Vision function	3.06*	**1.30**,** 7.21**	0.25	0.37, 1.67
Heart disease	1.03	0.75, 1.41	1.54*	**1.19**,** 1.99**
COPD	0.76	0.33, 1.77	2.11*	**1.34**,** 3.32**
Diabetes	1.66*	**1.12**,** 2.44**	1.85*	**1.30**,** 2.63**
Osteoporosis	0.42	0.11, 1.63	0.94	0.69, 1.28
Effects of a stroke	1.07	0.36, 3.21	1.42	0.51, 3.98
Mobility trouble	2.65*	**1.01**,** 6.95**	2.71*	**1.70**,** 4.31**

^∗^ *P* < 0.05.
